# Nonlinear association between blood lead and hyperhomocysteinemia among adults in the United States

**DOI:** 10.1038/s41598-020-74268-6

**Published:** 2020-10-13

**Authors:** Minghui Li, Lihua Hu, Wei Zhou, Tao Wang, Lingjuan Zhu, Zhenyu Zhai, Huihui Bao, Xiaoshu Cheng

**Affiliations:** 1 Department of Cardiovascular Medicine, The Second Affiliated Hospital of Nanchang University, No. 1 Minde Road, Nanchang, 330006 Jiangxi China; 2 Center for Prevention and Treatment of Cardiovascular Diseases, The Second Affiliated Hospital of Nanchang University, Nanchang, Jiangxi China

**Keywords:** Endocrine system and metabolic diseases, Metabolic disorders, Environmental sciences

## Abstract

Evidence regarding the association between blood lead levels (BLL) and hyperhomocysteinemia (HHcy) in US adults was limited. We aimed to investigate the association of BLL with the risk of HHcy, and to examine possible effect modifiers using US National Health and Nutrition Examination Survey (NHANES) database. We performed a cross-sectional study using data from up to 9,331 participants aged ≥ 20 years of NHANES from 2001 to 2006. BLL was measured by atomic absorption spectrometry. HHcy was defined as plasma homocysteine level > 15 µmol/L. The weighted prevalence of HHcy was 6.87%. The overall mean BLL was 1.9 μg/dL. Overall, there was a nonlinear positive association between Ln-transformed BLL (LnBLL) and the risk of HHcy. The Odds ratios (95% CI) for participants in the second (0.04–0.49 μg/dL), third (0.5–0.95 μg/dL) and fourth quartiles (> 0.95 μg/dL) were 1.12 (95% CI: 0.71, 1.76), 1.13 (95% CI: 0.73, 1.77), and 1.67 (95% CI: 1.07, 2.61), respectively, compared with those in quartile 1. Consistently, a significantly higher risk of HHcy (OR: 1.49; 95% CI: 1.19, 1.88) was found in participants in quartile 4 compared with those in quartiles 1–3. Furthermore, a strongly positive association between LnBLL and HHcy was observed in participants with estimated glomerular filtration rate (eGFR) < 60 mL/min^−1^/1.73 m^−2^. Our results suggested that a higher level of BLL (LnBLL > 0.95 μg/dL) was associated with increased risk of HHcy compared with a lower level of BLL (LnBLL ≤ 0.95 μg/dL) among U.S. adults, and the association was modified by the eGFR.

## Introduction

Hyperhomocysteinemia (HHcy), a metabolic disorder by an abnormally increased level of plasma homocysteine, is defined as plasma homocysteine level > 15 µmol/L^1^. HHcy is an established risk factor for a multitude of chronic conditions, such as Alzheimer’s disease, congestive heart failure, hearing loss, cancer, and atherosclerosis^2–6^. Although several factors have been demonstrated to be related to the prevalence of HHcy, including enzyme problems, lacking of cofactors, excessive methionine intake, certain diseases, and ingestion of certain drugs^7^. Increasing evidences have been suggested that heavy metals exposures may also lead to HHcy^8–10^. However, the potential impacts of heavy metals exposures have gained little attention.

Lead is a toxic metal widely distributed in the environment. In the past two decades, although regulatory actions had dramatically reduced lead exposures in the United States, it still occurs in many environmental settings, such as air pollution, smoking, contact with lead soils, certain foods, and drinking water^11–13^. Environmental low-dose Lead exposures also carried important public health implications^14,15^. Similarly to HHcy, a direct role of Lead was also observed in cardiovascular disease and cognitive dysfunction^16,17^. Given the similarities in these health effects, several previous studies have been examined the relationship between BLL and homocysteine^10,18–20^. Some epidemiologic studies had several strengths including prospective designs^18^. However, those studies have been limited by small sample sizes, lacking of adjustment for potential confounders, or other methodologic limitations. More importantly, few studies have thoroughly investigated the exact shape of the dose–response relationship between lead and HHcy. In addition, the possible modifiers in the association between lead and HHcy have not been comprehensively examined.

Therefore, our objective was to examine the association between blood lead and HHcy in a representative sample of U.S. adults aged ≥ 20 years who participated in the 2001–2006 National Health and Nutrition Examination Survey (NHANES). Additionally, we explored the possible effected modifiers in the association between BLL and HHcy.

## Methods

### Study population and design

The present study was performed using data from six years (2001–2006) of NHANES. Details of the NHANES had been described elsewhere^21,22^. In brief, NHANES used a complex multistage and stratified sampling design, and the participants were obtained from a representative of the noninstitutionalized civilian US population. During an interview at home, participants were invited to complete the questionnaires, perform medical examination, provide blood and other biological specimens in the mobile examination center. More detailed information is available on the official website (https://www.cdc.gov/nchs/nhanes/index.htm). The NHANES datasets were available on DataDryad (https://doi.org/10.5061/dryad.d5h62).

**Figure 1:**
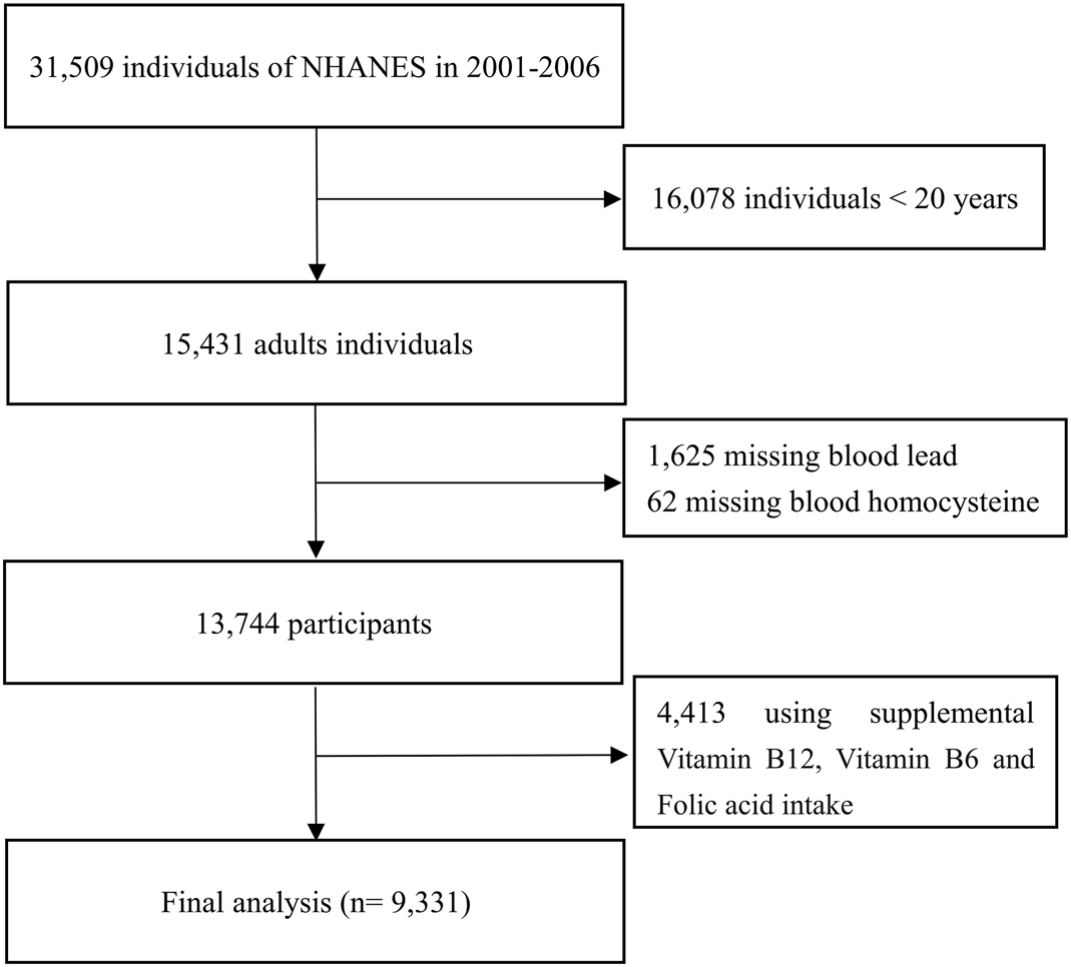
Flowchart of study participants. NHANES National Health and Nutrition Examination Survey 2001–2006.

A total of 15,431 adults aged ≥ 20 years were enrolled for NHANES 2001–2006. Excluded participants with missing blood lead (n = 1625) and missing blood homocysteine (n = 62), and those taking supplemental Vitamin B12, Vitamin B6 and Folic acid might affect homocysteine metabolism (n = 4413). Finally, a total of 9,331 subjects were included in our study (Fig. 1).

### Data collection procedures

Blood lead was measured at the Environmental Health Sciences Laboratory of the CDC National Center for Environmental Health (NCEH). Lead levels in whole blood were measured on a Perkin-Elmer model SIMAA 6000 (PerkinElmer, Norwalk, CT) simultaneous multielement atomic absorption spectrometer with Zeeman background correction, The limit of detection was 0.3 μg/dL in NHANES 2001–2004 and 0.25 μg/dL in NHANES 2005–2006. In present study, 0.53% and 0.24% of participants had BLL below the limits of detection in NHANES 2001–2004 and NHANES 2005–2006, respectively. Blood lead levels below the concentration of detection were assigned the limit of detection divided by the square root of 2 according to the recommendation of NHANES. National Institute of Standards and Technology whole-blood standard reference materials were used for external calibration. The interassay coefficients of variation ranged from 3.1% to 4.0% for blood lead.

The outcome variable was homocysteine. Homocysteine was measured by two methods, respectively, including Abbott Homocysteine IMX (HCY) assay (Abbott Diagnostics, Abbott Park, IL) in 2001 and Abbott Axsym system (Abbott Diagnostics, Abbott Park, IL) in 2002–2006. Long-term coefficients of variation for NHANES 2001–2006 were 3%-5% for total homocysteine concentrations. A crossover study was performed on the two methods, the results revealed no significant differences between two methods. Details of these assays and quality standards are available at https://cdc.gov/nchs/nhanes.

#### Other variables

Standard survey questionnaires were used to collect sociodemographic characteristics, including age, sex, race/ethnicity, marital status, education, physical activity, current smoking, alcohol intake and poverty income ratio. Race/ethnicity was defined as non-Hispanic white, non-Hispanic black, Mexican American, Other Hispanic and other races. Marital status was classified into married, single and living with partner. Educational background was grouped into less than high school, high school and above high school graduation. Physical activity was classified into sedentary, low, moderate and high. Current smoking was described yes or no. Alcohol intake was classified into low and high (< 3 and ≥ 3 drinks per day). Poverty income ratio was defined as the ratio of the midpoint of observed family income category to the official poverty threshold based on US Census Bureau information and classified into two groups (< 1 and ≥ 1). Physical examinations, such as weight and height were conducted following standardized protocol. Body mass index (BMI) was calculated as weight divided by height^2^ (kg/m^2^). Concentrations of cotinine were detected by using isotope dilution-high performance liquid chromatography/atmospheric pressure chemical ionization tandem mass spectrometry. Blood Cadmium levels were measured by using multielement atomic absorption spectrometer with Zeeman background correction. C-reactive protein (CRP) levels were measured by using latex-enhanced nephelometry on a Dade Behring Nephelometer II Analyzer System (BNII). Serum uric acid was measured on a Roche Hitachi Model 917 or 704 Multichannel Analyzer in 2001 and a Beckman Synchron LX20 in 2002 using a colorimetric method. Estimated glomerular filtration rate (eGFR) was calculated according to the Chronic Kidney Disease Epidemiology Collaboration (CKD-EPI) equation^23^. Both serum folate and vitamin B12 levels were determined by using the BioRad Laboratories “Quantaphase II Folate/Vitamin B12” radioassay kit (Bio-Rad Laboratories, Hercules, CA, USA). Dietary intake (Vitamin B12, Vitamin B6 and Folic acid) from foods was assessed using one in-person 24-h dietary recall during 2001–2002 and two 24-h dietary recalls (one in person and one 3–10 days later by telephone) for the 2003–2006 period. To calculate nutrient intake from foods, NHANES employed the US Department of Agriculture’s (USDA) Food and Nutrient Database for Dietary Studies (FNDDS) version 1 for NHANES 2001–2002, version 2 for NHANES 2003–2004, and version 3 for NHANES 2005–2006^24^.

### Statistical analysis

According to the guidelines of the CDC, two waves of continuous NHANES 2001–2002, 2003–2004 and 2005–2006 surveys were combined and six-year sampling weights were calculated. Because the distributions of blood lead levels (BLL) were highly skewed, the BLL was Ln-transformed (LnBLL) for analysis. We used weighted chi-square test (categorical variables) or weighted linear regression analyses (continuous variables) to calculate for differences among different LnBLL groups (quartiles). Data were presented as mean ± SD or proportions. We constructed multivariate logistic regression models to estimate odd ratios (ORs) for the risk of HHcy associated with LnBLL, after adjusting for the following covariates which were known as traditional or suspected risk factors for HHcy: Model I was adjusted for age, sex, BMI, race, education status, physical activity, marital status, poverty-to-income ratio, current smoking and alcohol intake. Model II was adjusted for all covariables in Model I plus adjusted for serum cotinine, blood cadmium, Serum Vitamin B12, Serum folate, Serum uric acid, eGFR, CRP, Vitamin B12 intake, Vitamin B6 intake and Folic acid intake. Additionally, to address for potential non-linear relationship between BLL and HHcy, a generalized additive model (GAM) and smooth curve fitting (penalized spline method) were conducted. Finally, to evaluate effect modification, the subgroup analyses were assessed for the variables: age, sex, BMI, race, education status, physical activity, marital status, current smoking, alcohol intake, eGFR.

We used the statistical package R software version 3.4.2 (https://www.R-project.org, The R Foundation) and Empower (R) version 2.17.8 (https://www.empowerstats.com; X&Y Solutions, Inc., Boston, MA) in the present analyses. A *P* value < 0.05 was considered statistically significant.

## Research ethics

NHANES study protocols were approved by the research ethics review board of the National Center for Health Statistics. Methods were carried out following the STROBE statement. Written informed consents were obtained from all participants in the study.

## Results

### Baseline characteristics of selected participants

Based on the inclusion and exclusion criteria, a total of 9,331 participants were enrolled, the mean age of the study participants was 44.4 years. Among the participants, 51.3% were men, 67.3% were Non-Hispanic White, 13.2% were Non-Hispanic Black, 9.4% were Mexican American, 4.9% were Other Hispanic, and 5.2% Other races. The mean concentrations of BLL and homocysteine were 1.9 μg/dL and 9.1 μmol/L, respectively. The prevalence of HHcy was 6.87%. The weighted distributions of population characteristics and other covariates according to LnBLL quartiles (< 0.04, 0.04–0.49, 0.5–0.95 and > 0.95 μg/dL) were shown in Table 1. There were significant differences between LnBLL quartiles, except for serum Vitamin B12 and Vitamin B6 intake. Participants in the highest quartile of LnBLL (BLL > 0.95 μg/dL) were older, more likely to be male, non-Hispanic black and Mexican American, to have lower poverty-to-income ratio, educational levels and physical activity, higher alcohol intake and lower folic acid intake, they also had higher values in homocysteine, blood cadmium, serum cotinine, serum uric acid and lower values in BMI, serum folate, eGFR and CRP than those of the other groups (all *P* < 0.05).

**Table 1:** Weighted characteristics of study population based on LnBLL quartiles. Abbreviations: BLL blood lead levels; BMI body mass index, eGFR estimated glomerular filtration rate, CRP C-reactive protein. *Data are presented as weighted means, proportions and Se. ¶The Physical activity categories were based on the distribution of MET-minute levels for the present NHANES sample. §BMI was calculated as the body weight in kilograms divided by the square of the height in meters. † Convert to BLL quartiles range: < 1.04 μg/dL, 1.04–1.64 μg/dL, 1.65–2.59 μg/dL, > 2.59 μg/dL.

Characteristic*	LnBLL quartiles†, μg/dL				
	Q1 (< 0.04)	Q2 (0.04–0.49)	Q3 (0.5–0.95)	Q4 (> 0.95)	*P *value
No of participants	2320	2336	2274	2401	
Age, years	35.5 ± 13.0	42.9 ± 15.7	49.5 ± 15.8	52.7 ± 16.7	< 0.001
Male, %	29.9	50.1	60.5	71.4	< 0.001
Race, %					< 0.001
Non-Hispanic White	67.6	68.5	69.6	62.6	
Non-Hispanic Black	13.0	13.3	11.1	16.0	
Mexican American	9.2	8.3	9.3	11.2	
Other Hispanic	6.1	4.5	4.4	4.4	
Other races	4.1	5.5	5.5	5.7	
Education, %					< 0.001
< High school	14.1	21.1	22.9	33.4	
High school	25.1	26.9	28.9	27.9	
> High school	60.8	52.0	48.2	38.8	
Marital status, %					< 0.001
Married	52.4	54.5	60.0	54.6	
Single	39.2	37.5	34.0	37.1	
Living with partner	8.3	8.0	6.0	8.3	
Physical activity, %¶					0.001
Sedentary	16.0	19.1	20.4	28.8	
Low	29.9	30.4	29.8	28.9	
Moderate	22.0	17.6	17.8	14.3	
High	32.0	32.8	32.0	28.1	
Poverty-to-income ratio	2.9 ± 1.6	2.9 ± 1.6	3.0 ± 1.6	2.5 ± 1.6	< 0.001
BMI, kg/m^2^	29.0 ± 7.6	28.7 ± 6.9	28.6 ± 6.1	27.5 ± 5.4	< 0.001
Current smoking,%	66.8	67.3	59.5	62.8	< 0.001
Alcohol intake					< 0.001
< 3 drinks per day	64.2	60.8	56.6	54.1	
≥ 3 drinks per day	35.8	39.2	43.4	45.9	
Laboratory data					
Homocysteine, μmol/L	7.4 ± 2.7	8.8 ± 3.9	9.8 ± 5.0	10.7 ± 5.1	< 0.001
Blood cadmium, μg/L	0.4 ± 0.4	0.6 ± 0.6	0.6 ± 0.7	0.9 ± 0.9	< 0.001
Serum cotinine, ng/mL	31.2 ± 86.9	74.2 ± 132.6	84.7 ± 143.0	118.6 ± 168.5	< 0.001
Serum Vitamin B12, pg/mL	492.5 ± 1004.5	477.2 ± 317.8	493.7 ± 869.5	520.5 ± 1069.8	0.429
Serum folate, ng/mL	12.1 ± 7.6	12.3 ± 17.8	11.8 ± 8.2	11.2 ± 7.9	0.017
Serum uric acid, mg/dL	4.9 ± 1.3	5.4 ± 1.4	5.7 ± 1.4	5.8 ± 1.4	< 0.001
eGFR, mL/min per 1.73 m^2^	99.6 ± 46.6	90.6 ± 21.5	86.3 ± 21.7	85.5 ± 24.5	< 0.001
CRP, mg/dL	0.5 ± 0.8	0.5 ± 1.1	0.4 ± 1.0	0.4 ± 0.7	0.002
Dietary					
Vitamin B12, μg/day	5.2 ± 4.6	5.4 ± 7.3	5.8 ± 7.4	5.8 ± 8.1	0.005
Vitamin B6, μg/day	1.9 ± 1.2	1.9 ± 1.2	2.0 ± 1.2	2.0 ± 1.1	0.174
Folic acid, μg/day	217.1 ± 196.9	194.3 ± 193.5	184.4 ± 154.6	180.2 ± 163.9	< 0.001

### Association of lead exposure with HHcy

**Figure 2:**
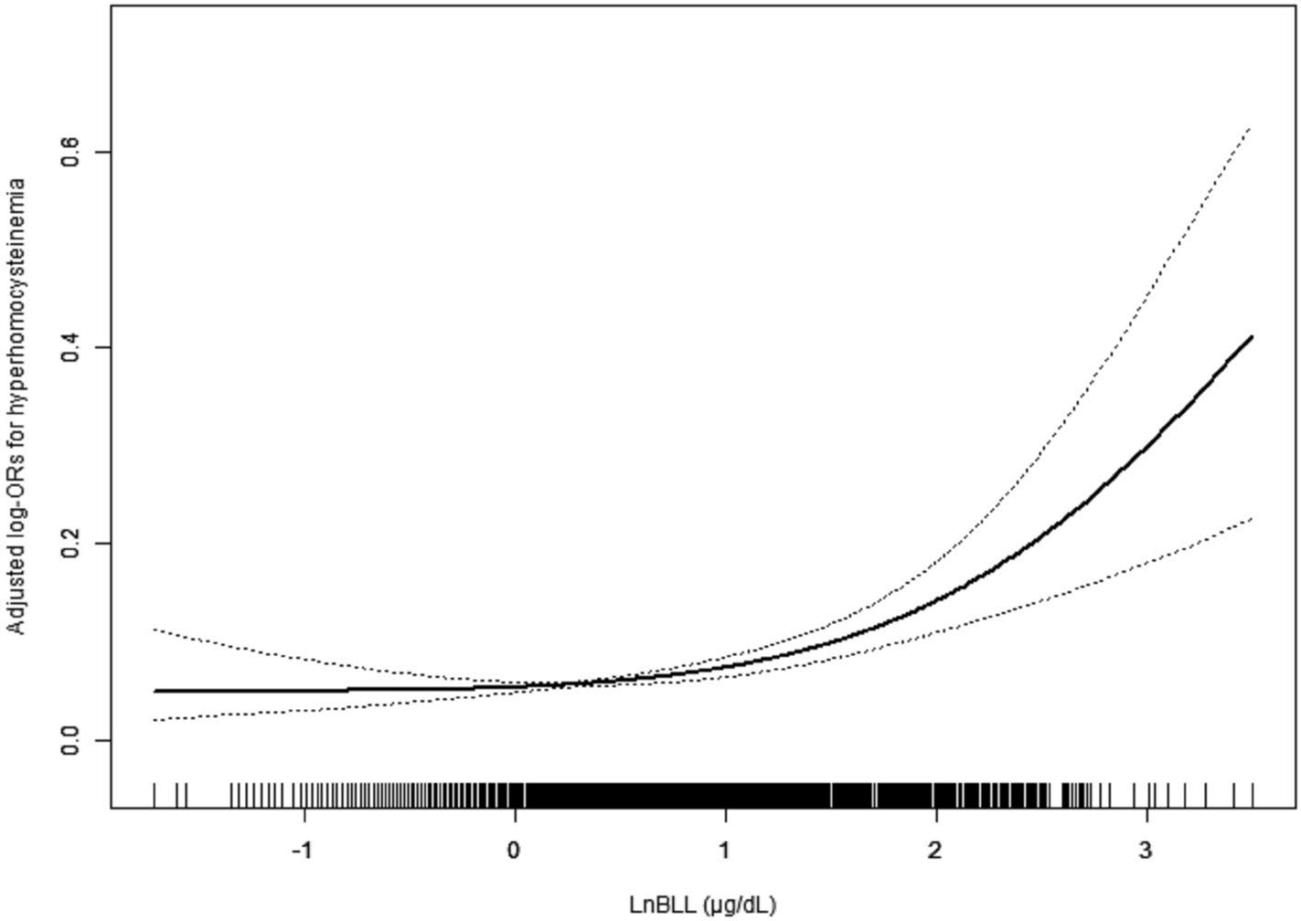
Dose–response relationship between LnBLL exposure and HHcy*. Abbreviations: BLL, blood lead level; HHcy, hyperhomocysteinemia; eGFR, estimated glomerular filtration rate. Solid line represents the smooth curve fit between variables. Dotted lines represent the 95 of confidence interval from the fit.*Adjusted for age, sex, BMI, race, education status, physical activity, marital status, poverty-to-income ratio, current smoking, alcohol intake, serum cotinine, blood cadmium, Serum Vitamin B12, Serum folate, Serum uric acid, eGFR, C-reactive protein, Vitamin B12 intake, Vitamin B6 intake and Folic acid intake.

The association between BLL and risk of HHcy was presented in Fig. 2. Overall, the risk of HHcy increased with BLL. The ORs and 95% CIs for these three equations were listed in Table 2. In the fully adjusted model (model II), when LnBLL was assessed as quartiles, compared with participants in quartile 1 of LnBLL, the adjusted ORs for participants in quartiles 2–4 were 1.12 (95% CI: 0.71, 1.76), 1.13 (95% CI: 0.73, 1.77), and 1.67 (95% CI: 1.07, 2.61), respectively. Consistently, a significantly higher risk of HHcy (OR: 1.49; 95% CI: 1.19, 1.88) was found in participants in quartile 4 compared with participants in quartiles 1–3. Conversely, a significantly lower risk of HHcy (OR: 0.67; 95% CI: 0.53, 0.84) was found in participants in quartiles 1–3 compared with participants in quartile 4. Additionally, multiple linear regression coefficients of homocysteine in association with LnBLL were presented in Supplementary Information Table 1, the results remained similar as in Table 2.

**Table 2:** Association between LnBLL and hyperhomocysteinemia in different models. Abbreviations: BLL blood lead levels. Crude model was adjusted for none. Model I: a*djusted for age, sex, BMI, race, education status, physical activity, marital status, poverty-to-income ratio, current smoking and alcohol intake.* Model II: *adjusted for all covariables in model 1 plus adjusted for serum cotinine, blood cadmium, Serum Vitamin B12, Serum folate, Serum uric acid, eGFR, C-reactive protein, Vitamin B12 intake, Vitamin B6 intake and Folic acid intake.*

LnBLL, μg/dL	Events (%)	Crude model		Model I		Model II	
		OR (95% CI)	*P* value	OR (95% CI)	*P *value	OR (95% CI)	*P *value
**Quartiles**							
Q1 (< 0.04)	36 (1.55)	Reference		Reference		Reference	
Q2 (0.04–0.49)	106 (4.54)	3.02 (2.06, 4.42)	< 0.001	1.59 (1.05, 2.40)	0.027	1.12 (0.71, 1.76)	0.617
Q3 (0.5–0.95)	158 (6.95)	4.74 (3.28, 6.84)	< 0.001	1.78 (1.19, 2.66)	0.005	1.13 (0.73, 1.77)	0.584
Q4 (> 0.95)	341 (14.20)	10.50 (7.41, 14.88)	< 0.001	2.81 (1.89, 4.19)	< 0.001	1.67 (1.07, 2.61)	0.025
**Categories**							
Q1–Q3 (≤ 0.95)	300(4.33)	Reference		Reference		Reference	
Q4 (> 0.95)	341(14.20)	3.66 (3.11, 4.31)	< 0.001	1.76 (1.44, 2.15)	< 0.001	1.49 (1.19, 1.88)	< 0.001
**Categories**							
Q4 (> 0.95)	341(14.20)	Reference		Reference		Reference	
Q1–Q3 (≤ 0.95)	300(4.33)	0.27 (0.23, 0.32)	< 0.001	0.57 (0.47, 0.70)	< 0.001	0.67 (0.53, 0.84)	< 0.001

### Stratified analysis by potential effect modifiers

**Figure 3:**
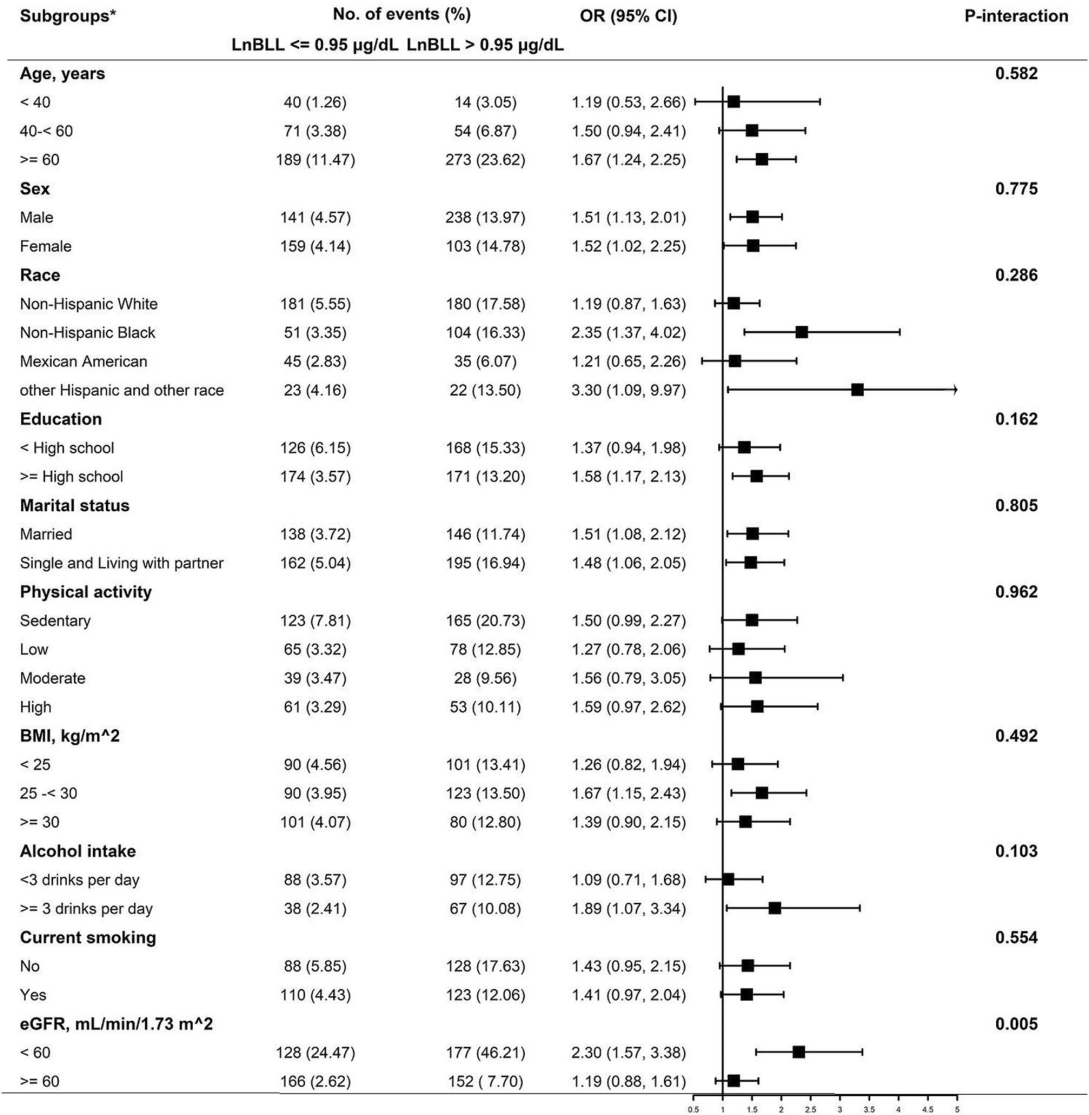
Subgroup analyses of the effect of LnBLL on HHcy. Abbreviations: BLL, blood lead levels; HHcy, hyperhomocysteinemia; BMI, body mass index; eGFR, estimated glomerular filtration rate; OR, odd ratios; CI, confidence interval. *Adjusted for age, sex, BMI, race, education status, physical activity, marital status, poverty-to-income ratio, current smoking, alcohol intake, serum cotinine, blood cadmium, Serum Vitamin B12, Serum folate, Serum uric acid, eGFR, C-reactive protein, Vitamin B12 intake, Vitamin B6 intake and Folic acid intake, except for the stratifying variable.

We further performed stratified analyses to assess the effect of LnBLL on HHcy in various subgroups. None of the variables, including age (< 40, 40- < 60, and ≥ 60 y; *P*-interaction = 0.582), sex (male and female; *P*-interaction = 0.775), race (Non-Hispanic White, Non-Hispanic Black, Mexican American and Other Race; *P*-interaction = 0.286), education (< High school and ≥ High school; *P*-interaction = 0.162), marital status (Married, Single and Living with partner; *P*-interaction = 0.805), physical activity (Sedentary, Low, Moderate and High; *P*-interaction = 0.962), BMI (< 25, 25–< 30 and ≥ 30 kg/m^2^; *P*-interaction = 0.492), alcohol intake (< 3 and ≥ 3 drinks per day; *P*-interaction = 0.103), and current smoking (no and yes; *P*-interaction = 0.554) significantly modified the association between LnBLL and HHcy (Fig. 3). However, there was a significant interaction between LnBLL and eGFR (< 60 and ≥ 60 mL/min^−1^/1.73 m^−2^) on HHcy. A stronger positive association between LnBLL and HHcy was found in participants with eGFR < 60 mL/min^−1^/1.73 m^−2^ (OR: 2.30; 95% CI: 1.57, 3.38) compared with eGFR ≥ 60 mL/min^−1^/1.73 m^−2^ (OR: 1.19; 95% CI: 0.88, 1.61; *P*-interaction = 0.005).

## Discussion

In this large cross-sectional study of a representative sample of U.S. adults who participated in NHANES 2001–2006, BLL was independently and positively associated with HHcy after adjusting other covariates. Moreover, we found a nonlinear relationship between BLL and HHcy. Higher LnBLL concentrations (> 0.95 μg/dL, quartile 4) was associated with a higher risk of HHcy compared with lower concentrations (≤ 0.95 μg/dL, quartiles 1–3). Furthermore, our study expanded the results of previous studies by demonstrating that the positive relation of BLL with HHcy was more pronounced in participants with eGFR < 60 mL/min^−1^/1.73 m^−2^.

The association between BLL and homocysteine had been examined in several previous studies^8,10,13,18,25,26^. In the United States, a sample of 1,140 adults who were 50–70 years of age, participated in the Baltimore Memory Study found a positive association of BLL with homocysteine, however, no association was found between tibia lead and homocysteine^10^. A prospective cohort study in 2301 American patients with a median follow-up of 3.9 y found that an interquartile range (IQR) increment in BLL (3 μg/dL) was associated with a 6.3% higher homocysteine concentration^18^. Guallar et al.^13^ performed a cross-sectional study among 4,447 persons aged ≥ 40 years who participated in the 1999–2002 NHANES and found that a positive linear association between BLL and homocysteine. However, our study found that the dose–response relationship between BLL and homocysteine was not linear (Supplementary Information Fig. 1) in the full adjusted model (model II). We speculated that several residual confoundings may conceal the true association between BLL and HHcy. For example, dietary intake and serum levels of folic acid, vitamin B12 and vitamin B6 status may be more likely to inflect homocysteine levels^27^; eGFR has been shown not only to increase homocysteine concentrations but also association with BLL^28,29^. Our results agreed with earlier reports in other NHANES (2003–2004) study^20^, which revealed a nonlinear increase in the risk of HHcy prevalence with increase BLL. This study found that, in the fully adjusted models compared with participants in quartile 1 (BLL ≤ 1.0 μg/dL), the significant increased risk of HHcy was found in participants in quartile 4–3 and 4–4 (BLL ≥ 3.5 μg/dL; OR: 2.6; 95% CI: 1.4, 4.8 and 3.2; 95% CI: 1.7, 5.9, respectively), not in quartile 2 to quartile 4–2.

The pathophysiology of the association between BLL and homocysteine has not yet been totally explicated. A primary mechanism for lead toxicity is inhibition heme and hemoglobin synthesis^30^, thus directly affecting the function of cystathionine β-synthase, which is a unique heme-containing in transsulfuration to cysteine of homocysteine, leading to an accumulation of homocysteine^31^. Additionally, BLL has high electron-sharing affinities for the free sulfhydryI group of proteins, homocysteine itself also contains a sulfhydryl group, so BLL may inhibit the metabolism of homocysteine by this pathway^30,32^. Moreover, in the remethylation pathway of homocysteine through the enzyme methionine synthase with vitamin B12, folate, and MTHFR, when the MTHFR enzyme mutates might cause Lead to bind more to active sites, and cause impaired enzyme function^33^.

In the present study, a strong association between BLL and HHcy was found in participants with low eGFR levels (< 60 mL/min^−1^/1.73 m^2^). Chronic lead exposure may contribute to chronic kidney disease, indicated by low eGFR^34,35^. In the Malmö Diet and Cancer Study-Cardiovascular Cohort, for example, BLL has been shown to decrease eGFR in participants of the prospective study aged 46 to 67 years^34^. Similarly to lead, elevated homocysteine level was also associated with an accelerated decline in renal function in the general population^36^. Conversely, a inverse relationship between the eGFR and plasma homocysteine level was present throughout the whole range of renal function^37^. Therefore, eGFR may modify the relation between BLL and homocysteine. However, the causal mechanisms are not fully understood, further studies focused on the suggested pathway are warranted.

### Study limitations

There were several limitations in our study. Firstly, taking into account the present study was a cross-sectional study, the directionality of the association between BLL and HHcy cannot be conclusively established. Although we made an effort to adjust for as many potential confounders as possible, bias due to unmeasured confounding may still remain. Secondly, the biologic residence time of Lead in blood was measured in few days, single BLL may be limited to assess the association between BLL and HHcy. However, it has been reported that BLL can be considered as a steady-state mixture from both acute external Lead exposure and the internal stores of Lead, and can be associated with plasma homocysteine levels 4–6 years later^25,38^. Thirdly, the gene interaction could not be considered in present study, which was already known to be associated with the level of homocysteine (e.g., MTHFR gene). Further studies with different genotypes related to Lead and homocysteine metabolism are warranted to better investigate the association between Lead exposure and HHcy.

## Conclusions

Our study revealed a nonlinear association between BLL and HHcy in a U.S. adults, and the association was modified by the eGFR. Additional studies are warranted to confirm our findings in prospective cohorts and to elucidate the potential mechanisms underlying the relationship between Lead and HHcy.

## Supplementary information

Supplementary Information
