# Supplementary material for: Nonlinear association between blood lead and hyperhomocysteinemia among adults in the United States
Source: Sci Rep. 2020 Oct 13;10:17166. doi: 10.1038/s41598-020-74268-6 (PMC7553908; doi:10.1038/s41598-020-74268-6)
Supplement: Supplementary file 1 — Supplementary Information [file 41598_2020_74268_MOESM1_ESM.docx]

**Nonlinear association between blood lead and hyperhomocysteinemia among adults in the United States**

Minghui Li^1^, Lihua Hu^1^, Wei Zhou^2^, Tao Wang^2^, Lingjuan Zhu^2^, Zhenyu Zhai^1^, Huihui Bao^1,2*^, Xiaoshu Cheng^1,2*^

^1^Department of Cardiovascular Medicine, the Second Affiliated Hospital of Nanchang University, Nanchang of Jiangxi, China.

^2^Center for Prevention and Treatment of Cardiovascular Diseases, the Second Affiliated Hospital of Nanchang University, Nanchang of Jiangxi, China.

*Correspondence and reprint requests should be addressed to:

Huihui Bao, M.D., Ph.D.

Department of Cardiovascular Medicine, the Second Affiliated Hospital of Nanchang University, Nanchang of Jiangxi, China.No. 1 Minde Road, Nanchang of Jiangxi, 330006, China.

Email: huihui_bao77@126.com

Phone: +8613870092915

Fax: 0086-0791-86262262

or

Xiaoshu Cheng, M.D., Ph.D.

Department of Cardiovascular Medicine, the Second Affiliated Hospital of Nanchang University, Nanchangof Jiangxi, China.No. 1 Minde Road, Nanchang of Jiangxi, 330006, China.

Email: xiaoshumenfan126@163.com

Phone: +8613607089128

Fax: 0086-0791-86262262


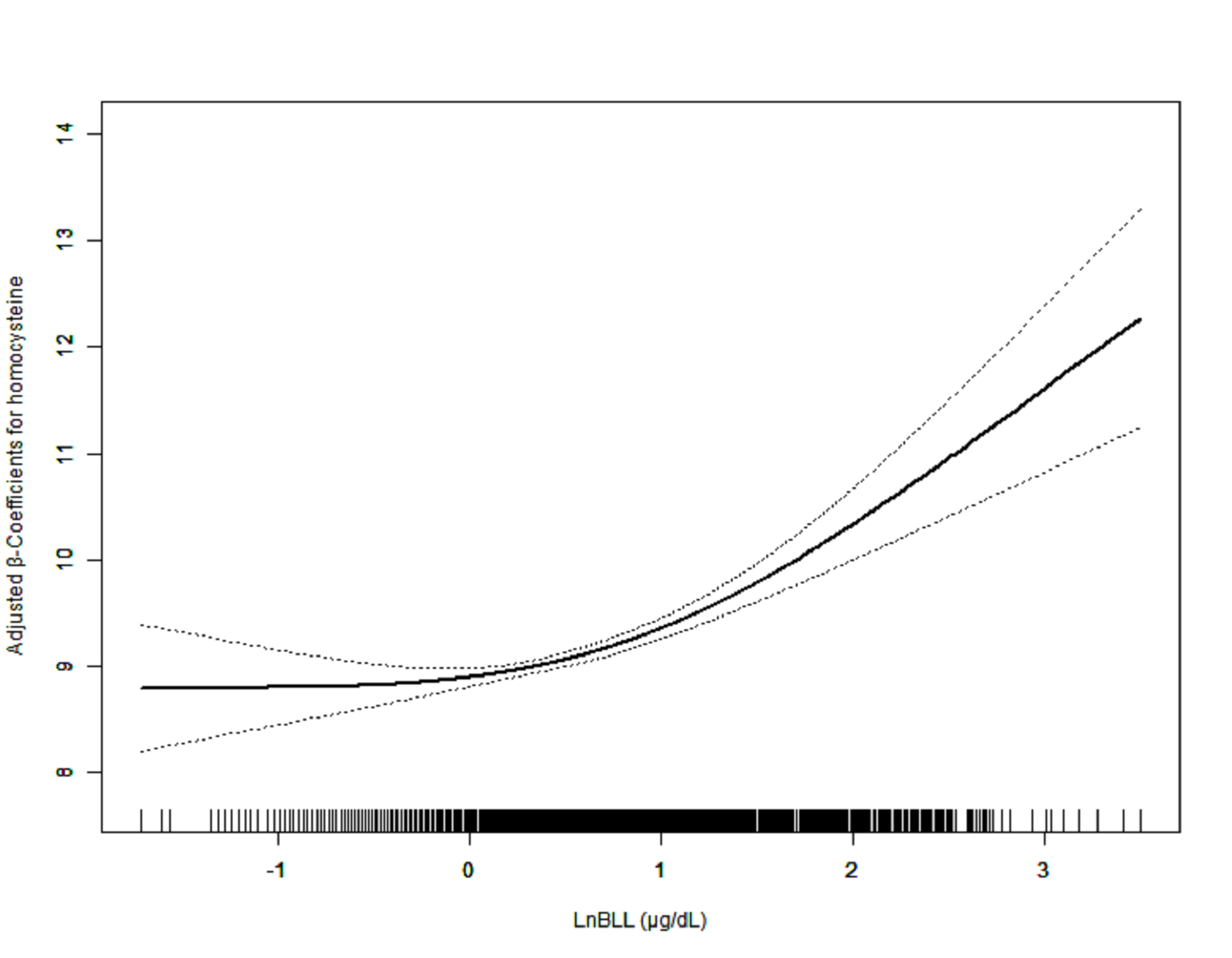


**Supplemental Figure 1**. Dose–response relationship between LnBLL exposure and homocysteine*. Abbreviations: BLL, blood lead level; eGFR, estimated glomerular filtration rate. Solid line represents the smooth curve fit between variables. Dotted lines represent the 95 of confidence interval from the fit.**Adjusted for age, sex, BMI, race, education status, physical activity, marital status, poverty-to-income ratio, current smoking, alcohol intake, serum cotinine, blood cadmium, Serum Vitamin B12, Serum folate, Serum uric acid, eGFR, C-reactive protein, Vitamin B12 intake, Vitamin B6 intake and Folic acid intake.*

| LnBLL, μg/dL | Crude Model | | Model Ⅰ | | Model Ⅱ | | |
| --- | --- | --- | --- | --- | --- | --- | --- |
|  | β (95%CI) | P-value | β (95%CI) | P-value | | β (95%CI) | P-value |
| Per 1 μg/dL increase | 2.19 (2.06, 2.33) | <0.001 | 0.82 (0.65, 0.99) | <0.001 | | 0.53 (0.35, 0.70) | <0.001 |
| Quartiles |  |  |  |  | |  |  |
| Q1 (< 0.04) | Reference |  | Reference |  | | Reference |  |
| Q2 (0.04-0.49) | 1.63 (1.36, 1.90) | <0.001 | 0.45 (0.17, 0.73) | 0.001 | | 0.13 (-0.15, 0.41) | 0.357 |
| Q3 (0.5-0.95) | 2.64 (2.37, 2.92) | <0.001 | 0.73 (0.43, 1.02) | <0.001 | | 0.31 (0.01, 0.62) | 0.043 |
| Q4 (> 0.95) | 3.94 (3.67, 4.21) | <0.001 | 1.29 (0.97, 1.60) | <0.001 | | 0.77 (0.44, 1.10) | <0.001 |
| Categories |  |  |  |  | |  |  |
| Q1-Q3 (≤ 0.95) | Reference |  | Reference |  | | Reference |  |
| Q4 (> 0.95) | 2.52 (2.30, 2.75) | <0.001 | 0.78 (0.55, 1.02) | <0.001 | | 0.58 (0.33, 0.82) | <0.001 |

**Supplemental Table 1.** Relationship between LnBLL and homocysteine in different models. Crude model was adjusted for none. Model I: *adjusted for age, sex, BMI, race, education status, physical activity, marital status, poverty-to-income ratio, current smoking and alcohol intake.* Model II: *adjusted for all covariables in model 1 plus adjusted for serum cotinine, blood cadmium, Serum Vitamin B12, Serum folate, Serum uric acid, eGFR, C-reactive protein, Vitamin B12 intake, Vitamin B6 intake and Folic acid intake.*
